# Predicting individual food valuation via vision-language embedding model

**DOI:** 10.1371/journal.pdig.0001044

**Published:** 2025-10-28

**Authors:** Hiroki Kojima, Asako Toyama, Shinsuke Suzuki, Yuichi Yamashita

**Affiliations:** 1 Department of Information Medicine, National Institute of Neuroscience, National Center of Neurology and Psychiatry, Kodaira, Tokyo, Japan; 2 Hitotsubashi Institute for Advanced Study, Hitotsubashi University, Kunitachi, Tokyo, Japan; 3 Graduate School of the Humanities, Senshu University, Kawasaki, Kanagawa, Japan; 4 Faculty of Social Data Science, Hitotsubashi University, Kunitachi, Tokyo, Japan; 5 Centre for Brain, Mind and Markets, The University of Melbourne, Melbourne, Australia; Mayo Clinic Rochester: Mayo Clinic Minnesota, UNITED STATES OF AMERICA

## Abstract

Food preferences differ among individuals, and these variations reflect underlying personalities or mental tendencies. However, capturing and predicting these individual differences remains challenging. Here, we propose a novel method to predict individual food preferences by using CLIP (Contrastive Language-Image Pre-Training), which can capture both visual and semantic features of food images. By applying this method to food image rating data obtained from human subjects, we demonstrated our method’s prediction capability, which achieved better scores compared to methods using pixel-based embeddings or label text-based embeddings. Our method can also be used to characterize individual traits as characteristic vectors in the embedding space. By analyzing these individual trait vectors, we captured the tendency of the trait vectors of the high picky-eater group. In contrast, the group with relatively high levels of general psychopathology did not show any bias in the distribution of trait vectors, but their preferences were significantly less well-represented by a single trait vector for each individual. Our results demonstrate that CLIP embeddings, which integrate both visual and semantic features, not only effectively predict food image preferences but also provide valuable representations of individual trait characteristics, suggesting potential applications for understanding and addressing food preference patterns in both research and clinical contexts.

## Author summary

Food preferences vary greatly among individuals and can provide insights into personality traits and mental health patterns. Traditional approaches to understanding these preferences have been limited by their inability to capture the complex interplay between what we see and what we know about food. In this study, we developed a new computational method using CLIP (Contrastive Language-Image Pre-Training), an artificial intelligence model that can analyze both visual features and semantic meaning simultaneously. We tested our approach on food rating data from 199 participants who evaluated 896 food images. Our method successfully predicted individual food preferences and revealed distinct patterns in people with different eating behaviors and mental health characteristics. Notably, individuals with picky eating tendencies showed preference patterns that systematically avoided healthy foods, while those with higher mental health symptom scores had less consistent preference patterns overall. These findings demonstrate that combining visual and semantic information provides a powerful tool for understanding food preferences, with potential applications in personalized nutrition, clinical assessment, and treatment of eating disorders.

## Introduction

Food choices and preferences play crucial roles not only in our daily lives but also in medical contexts, particularly in relation to eating disorders such as selective eating and food avoidance [1]. Understanding and predicting food preferences has significant implications for both public health interventions and clinical applications [2]. Recent advances in personalized nutrition have demonstrated the clinical utility of individualized dietary recommendations based on genetic, metabolic, and behavioral factors [3,4]. Furthermore, computational approaches to food preference modeling have shown promise in clinical settings, including automated dietary assessment for managing diabetes [5], personalized interventions for eating disorders [6], and precision nutrition strategies for metabolic syndrome prevention [7]. More recently, machine learning applications in nutrition research have expanded significantly [8], with artificial intelligence demonstrating potential for deciphering complex diet-disease relationships [9], highlighting the growing need for objective, scalable methods to characterize individual food preference patterns in clinical contexts.

A distinctive feature of food preference as a research target is its multifaceted nature [10,11]. When we judge an apple as appetizing, for instance, we process various modalities of information: semantic information (recognition that it is an apple), visual features (such as its redness), olfactory cues, tactile sensations like weight, and more [12–16]. Furthermore, while general trends in food preferences exist, significant individual variations pose additional challenges to understanding and predicting these preferences [17]. However, existing models often fall short in capturing the complex interplay between these facets at an individual level, necessitating more advanced computational approaches.

In this study, we focus on vision among various modalities, which has been identified as one of the most influential modalities in food preference [18,19]. Traditional approaches to food preference prediction have often relied on categorical or demographic features [20], but recent advances in computer vision and neural networks offer new opportunities for capturing subtle visual characteristics that influence preference.

Furthermore, a critical consideration for food preference prediction is that both visual and semantic information play essential and interactive roles in how we evaluate food items [12,21]. Experimental studies show that identical foods receive different evaluations when labeled differently [22,23], while the same food item can receive different responses when its visual appearance is altered [24,25]. This integrated nature of food perception suggests that preference prediction should capture the interaction between visual and semantic features, rather than treating them as separate components. Building on these insights and recent computer vision techniques, in this study, we develop and validate a more comprehensive food preference prediction model that integrates both visual and semantic information.

At the core of our methodology are embedding techniques, which convert data into vector representations. The significance of these techniques lies in their ability to establish distances or similarities between data points [26]. For instance, someone who enjoys cake might likely appreciate similar sweet baked goods like muffins or cookies, while their preference for more dissimilar foods like broccoli or grilled fish may be harder to predict. Finding appropriate similarity metrics is thus crucial for prediction, and embeddings enable this by preserving relevant distance relationships from the original data space.

Importantly, similarity relationships are not uniquely determined. For example, while photos of red and green apples might have high semantic similarity (both being apples), their visual similarity might be relatively low due to color differences. Different embedding approaches can capture these distinct types of similarities: pixel-based embeddings reflect visual distances, while semantic label embeddings capture meaning-based relationships [27].

In this study, we primarily utilize CLIP [28] as an embedding technique that simultaneously embeds both images and text. This is based on the assumption that both semantic and visual elements contribute to preferences, and their interaction may be complex rather than merely additive. This is analogous to cognitive effects like the Stroop effect, where semantic and visual features interact and interfere with each other [29]. By training on paired image-text data, CLIP’s embedding structure is expected to reflect both visual and semantic similarities, making it particularly suitable for food preference prediction. Indeed, recent research by Shoham et al. [30] on mental representations of familiar faces and objects has provided strong evidence that human visual and semantic processing may be more integrated than previously thought. Their work has demonstrated significant correlations between CLIP-derived similarities and human perceptual judgments, particularly in tasks involving visual object recognition and categorization. Their findings suggest that CLIP’s representations capture some aspects of human visual processing, with especially strong correspondences in cases where visual features dominate semantic influences.

While Shoham et al. [30] demonstrated CLIP’s ability to capture human-like visual-semantic representations in object recognition and memory tasks, our study extends this foundation to the specific domain of food preference prediction with several novel contributions. First, we apply vision-language embeddings to food images, where the integration of visual and semantic information is particularly critical for human evaluation processes. Second, we advance beyond population-level analysis to characterize individual preference patterns through trait vectors in embedding space, enabling personalized prediction of individual food preferences. Third, we relate the obtained individual trait vectors to psychological factors, including mental health symptom patterns and picky eating tendencies, demonstrating the clinical relevance of vision-language embedding approaches.

This approach differs fundamentally from previous food-related studies that have focused primarily on classification or recognition tasks [31,32], instead leveraging CLIP’s pre-trained vision-language alignments to model the complex, subjective nature of human food preference formation.

We compare this approach with other image-based embeddings (pixel embeddings) and text-based embeddings (OpenAI embeddings) to evaluate its effectiveness. The image embeddings, which use the distance of pixel values, provide a pure image similarity baseline. In contrast, text embeddings using the label information–that is, the food names provided as annotations (e.g., cheese burger, apple, cake)–offers semantic representations derived purely from textual descriptions.

The experimental data we analyze include 199 subjects’ ratings (tasty, favorite, healthy) of food images in the Food-Pics_Extended [33]. This dataset is particularly valuable as it captures both semantic information about the food items and their specific visual characteristics. Our study aims to predict not only the average ratings across subjects but also individual ratings, providing insights into personal preference patterns and their potential relationship with eating behaviors and psychological factors [34].

In summary, this research analyzes subject ratings of food images using CLIP embeddings, aiming to predict both average and individual ratings. We further analyze individual characteristics derived from these predictions, examining their relationships with selective eating tendencies and mental health indicators [35,36]. This work contributes to our understanding of how visual and semantic features interact in food preference formation and offers potential applications in both clinical and everyday contexts.

## Methods

### Rating experiments

Data collection was conducted through a web survey on individual differences in value judgments towards food images using the Qualtrics Survey Software (Qualtrics, Provo, UT). Initial recruitment through the online crowdsourcing service CrowdWorks Inc. (Japan) yielded 247 participants, of whom 48 were excluded based on predetermined criteria: incomplete survey responses, failed attention checks, and age outside the target range. The final sample comprised 199 participants (90 males and 109 females, mean age 39.0 years, SD = 11.4) who were native Japanese speakers aged 20 years or older.

Participants were instructed to rate 896 food images from the Food-Pics_Extended dataset [33] on an 8-point Likert scale ranging from 1 (strongly disagree) to 8 (strongly agree) for three questions: (1) “Do you like the food?"; (2) “Is the food tasty?"; and (3) “Is the food healthy?”. To minimize fatigue effects and ensure data quality, the experiment was completed over three separate days within a ten-day period. On each day, participants rated the food images on one of the three dimensions, with the order randomized across participants. At the end of each experimental session, participants also rated the overall familiarity of the presented food images.

To minimize the potential effects of homeostatic mechanisms, such as satiation, participants were instructed to refrain from eating or drinking anything besides water for 3 hours before each experimental session [14,37]. Data quality was ensured through embedded attention checks throughout the survey, and completion rates exceeded 95% for participants who began the actual rating task.

In addition to food ratings, participants completed questionnaires on the first day including the Unbalanced Diet Scale (measuring picky eating) [38] and the DSM-5 Level 1 Cross-Cutting Symptom Measure (assessing overall mental-health status) [39]. The experimental protocol was approved by the Ethics Committee of National Center of Neurology and Psychiatry (A2021-072), and participants provided informed consent online after reading the description displayed on screen. Complete methodological details and summary statistics are available in a separate preprint [40].

### Embedding food images

To embed food images into vector space, we used CLIP [28]. Throughout this paper, “CLIP” denotes the CLIP model with a ViT-B/16 backbone (CLIP-ViT) unless otherwise noted. As a preprocessing step, each image was coarse-grained and resized to 224×224 pixels to match CLIP’s input specifications. For visualization purposes, we further embedded these CLIP vectors onto two-dimensional UMAP space using standard parameters (n_neighbors=15, min_dist=0.1). UMAP was chosen for its ability to preserve local neighborhood structure while maintaining interpretable global organization [41]. All quantitative analyses were performed on the original CLIP embeddings; UMAP was used solely for visualization and does not affect computational results.

For comparison, we also employed two alternative embedding approaches (PIXEL-Emb, TEXT-Emb), in addition to CLIP embeddings (CLIP-Emb).

- CLIP Embedding (CLIP-Emb): Images were preprocessed to 224×224 pixels and embedded into CLIP vectors.
- Pixel-based UMAP (PIXEL-Emb): Images were preprocessed following the same procedure as CLIP, and a distance matrix was constructed using Euclidean distance between pixel values.
- Text embedding (TEXT-Emb): Using OpenAI’s embeddings model (’text-embedding-ada-002’), we embedded the food category labels from the Food-Pics_Extended dataset.

### Average rating prediction using CLIP

The prediction of average ratings utilized ridge regression based on CLIP embeddings. The model is formulated as:


$$\hat{y}=X\beta$$

where $\hat{y}$ represents the predicted average ratings, and *X* is the matrix of image embedding vectors. The objective function minimized in ridge regression is given by:


$$\min_{\beta }||y-X\beta |{|}^{2}+\lambda ||\beta |{|}^{2}$$

where *y* represents the average rating from the data, λ is the regularization parameter, and $||\beta |{|}^{2}$ is the L2 norm of the regression coefficients. Given our moderate dataset size (n = 199) and the subsequent use of regression coefficients as individual trait vectors for psychological analysis, we selected λ=1.0 as a conservative regularization parameter. Preliminary analysis across different *λ* values using our 5-fold cross-validation framework showed peak test performance at λ=0.1, with the one-standard-error rule suggesting λ≈0.7, but we prioritized coefficient stability over marginal prediction gains to ensure robust trait vector extraction (see S1 Text for details).

To ensure robust evaluation of prediction performance, we employed a 5-fold cross-validation approach. Model performance was evaluated using both Mean Squared Error (MSE) and Pearson correlation coefficients. While MSE provides a direct measure of prediction accuracy in the original rating scale, correlation coefficients capture the model’s ability to preserve preference ordering. Both metrics were calculated for each fold to assess model performance across different data subsets.

To validate our methodological choice, we conducted a comparative analysis with established vision architectures including ResNet-101 (supervised, ImageNet-1k) [42,43], ViT-B/16 (supervised, ImageNet-21k) [43,44], EfficientNet-B0 (supervised, ImageNet-1k) [43,45], and CLIP-ResNet (ResNet-101 backbone; CLIP contrastive pretraining) [28,42]. The CLIP model adopted in this study (CLIP-ViT) consistently outperformed all comparison methods across rating dimensions (see S1 Text for detailed results), confirming its suitability for food preference prediction.

### Simulation of subjective ratings with CLIP

The linear fitting of *y* shown above ($\hat{y}=X\beta$) can be interpreted as computing the cosine similarity between *β* and image embedding vectors, up to a scaling factor.

Here, instead of finding this *β* by fitting the training data, we also checked the prediction performance directly using the embedding vectors obtained from converting corresponding questionnaire text using CLIP, which corresponds to directly “asking” CLIP the corresponding questions (S6 Fig). The actual procedures are as follows:

1. Embedding the questions “This is a tasty food”, “This is my favorite food” and “This is a healthy food” into CLIP vectors respectively.

2. Calculating the cosine similarity between these query vectors and food image vectors from the dataset. We assumed that this similarity corresponds to the rating for each item.

3. Comparing the obtained cosine similarity and mean rating from subjective experiment using correlation coefficient analysis.

### Prediction of individual preferences

Based on these embeddings, we predicted each individual’s rating (“like”, “tastiness”, and “healthiness”) using ridge regression. The model is formulated as:


$$\hat{y_{i}}=X\beta_{i}$$

where $\hat{y_{i}}$ represents the predicted rating of the subject *i*, and *X* is the matrix of image embedding vectors. The objective function minimized in ridge regression is given by:


$$\min_{\beta }||y_{i}-X\beta_{i}|{|}^{2}+\lambda ||\beta_{i}|{|}^{2}$$

Here, *y*_*i*_ is the vector of observed ratings of the subject *i*, λ is the regularization parameter, and $||\beta_{i}|{|}^{2}$ is the L2 norm of the regression coefficients. The regression coefficients $\beta_{i}$ obtained from this process for subject *i* will be used for the characterization of each subject. Following the average rating prediction, we employed the same regularization parameter (λ=1.0) for individual preference modeling.

Performance evaluation followed the same 5-fold cross-validation procedure used in average rating prediction, with separate models fitted for each individual. The obtained trait vectors were used to characterize individual preferences in subsequent analyses.

#### Characterization of individual traits.

To characterize individual differences beyond prediction accuracy, we utilized the regression coefficients obtained from the model fitting process. Specifically, for each subject *i*, the regression coefficient vector, $\beta_{i}$ obtained from fitting the complete dataset was defined as their individual trait vector $x_{i}^{trait}$. This vector represents the subject’s unique pattern of food preferences in the embedding space.

Additionally, we quantified how well the model captured each individual’s preference pattern by calculating the correlation coefficient (*r*_*i*_) between the predicted ratings $\hat{y_{i}}$ and observed ratings *y*_*i*_ for each subject. Unlike the cross-validation procedure used for prediction performance evaluation, this characterization analysis utilized coefficients from fitting the entire dataset to obtain the most stable representation of individual traits.

## Results

### CLIP embeddings and their characteristics

**Fig 1:**
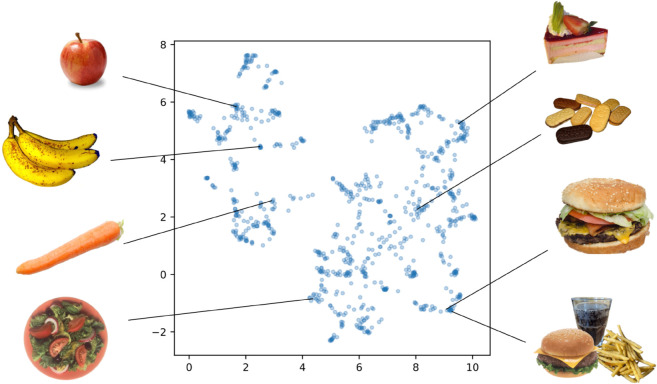
Embedding obtained by CLIP and example of corresponding food images (public-domain replacements; originals: Food_Pics_Extended IDs 0796, 0798, 0208, 0196, 0028, 0164, 0002, 0003).

First, we embedded the food image dataset using CLIP (CLIP-Emb) and visualized the resulting vectors via UMAP dimension reduction (Fig 1). In this figure, each food image was represented as a data point in the UMAP space, positioned reflecting the similarity in CLIP embeddings, which was also consistent with our intuitive categories. Furthermore, the distribution of the data points was not uniform and some internal structures were observed. By using the food category annotation provided in the original dataset, we confirmed that these structures align with some food categories, such as whole foods/ processed foods, fruit, fish and drinks (S2 Fig).

We also generated embeddings using different methods, pixel-based embeddings and label embeddings, PIXEL-Emb, TEXT-Emb. The UMAP visualization of these embeddings were shown in S3 Fig. Especially, in the pixel-based embeddings, the obtained data points were uniformly distributed and no apparent internal structure was observed. To quantitatively measure the similarity of these embeddings and CLIP-Emb, we calculated the correlation coefficient between the distance matrix of the embeddings (S1 Fig). We found that CLIP-Emb and TEXT-Emb showed moderate correlation (r = 0.49), while PIXEL-Emb showed minimal correlation with both (r = 0.15, 0.10 for CLIP-Emb and TEXT-Emb, respectively). The moderate correlation between CLIP-Emb and TEXT-Emb, in contrast to PIXEL-Emb’s minimal correlation with both, suggests that semantic information similar to that in text embeddings is more strongly reflected in CLIP-Emb compared to the pixel-level visual features captured in PIXEL-Emb.

On the other hand, CLIP-Emb retained some information of visual features. For example, the “dark brownness” of the cookie can be extracted from CLIP-Emb (S4 Fig), which cannot be distinguished by TEXT-Emb because they have the same label.

### Prediction of mean ratings using CLIP embeddings

**Fig 2:**
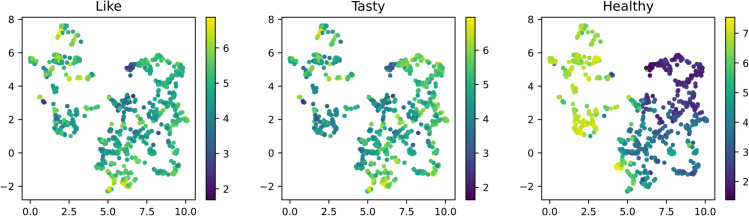
Mean ratings from the web experiment for ’Like’ (left), ’Tasty’ (middle), and ’Healthy’ (right) dimensions, visualized on the UMAP projection of CLIP embeddings. Each point represents a food item, with color intensity indicating the magnitude of the average rating.

We analyzed the rating data (“like”, “tasty” and “healthiness”) obtained from the subject experiment using the food image embeddings. In this section, we first used the averaged rating of each food image over all subjects. We visualized the average rating on the CLIP-Emb (Fig 2) and the other embeddings, PIXEL-Emb, TEXT-Emb (S5 Fig). We observed that the ratings showed systematic patterns in both CLIP space and label space, while appearing relatively random in the PIXEL-Emb. This suggests that these embeddings, particularly CLIP-Emb and TEXT-Emb, were predictive of subjective ratings.

We estimated this predictive capability of these mean ratings by implementing prediction model using ridge regression. Using 5-fold cross-validation, our proposed regression model with CLIP-Emb achieved an average MSE of $3.9\;\pm \;0.2\;\times \;10^{-2}$ (“like”), $4.1\;\pm \;0.2\;\times \;10^{-2}$ (“tasty”), and $4.8\;\pm \;0.2\;\times \;10^{-2}$ (“healthy”) and an average correlation coefficient of 0.771±0.019 (“like”), 0.792±0.014 (“tasty”), and 0.944±0.006 (“healthy”). A lower MSE indicates that the predicted values are, on average, close to the actual values, reflecting small overall errors. The correlation coefficient further demonstrates the linear relationship between predictions and true values, where a value close to 1.0 implies a strong positive correlation. Together, these metrics suggest that our model reliably captures the underlying patterns in the data.

From the TEXT-Emb, we obtained average MSE of $4.5\;\pm \;0.3\;\times \;10^{-2}$ (“like”), $4.9\;\pm \;0.3\;\times \;10^{-2}$ (“tasty”), and $5.6\;\pm \;0.1\;\times \;10^{-2}$ (“healthy”) and an average correlation coefficient of 0.694±0.043 (“like”), 0.710±0.052 (“tasty”), and 0.926±0.008 (“healthy”), and from the PIXEL-Emb, we obtained average MSE of $6.0\;\pm \;0.2\;\times \;10^{-2}$ (“like”), $6.3\;\pm \;0.2\;\times \;10^{-2}$ (“tasty”), and $1.39\;\pm \;0.05\;\times \;10^{-1}$ (“healthy”) and an average correlation coefficient of 0.189±0.040 (“like”), 0.302±0.029 (“tasty”), and 0.179±0.043 (“healthy”). From these results, we confirmed that the predictive power of CLIP-Emb yielded better scores compared to TEXT-Emb and PIXEL-Emb.

#### Zero-shot prediction using text query.

The prediction system in the previous section corresponds to finding an appropriate *β* by fitting that satisfies y≃Xβ, where *y* is the average rating and *X* is the matrix of food image embeddings. We note here that Xβ can be interpreted as the cosine similarity between *β* and each image embedding, up to a multiplicative factor.

Here, instead of finding *β* by ridge regression, we explored whether zero-shot prediction is possible using the characteristics of CLIP, which can embed both images and text. The strategy here is to directly “ask” the same rating question to CLIP. We encoded the corresponding texts (“This is my favorite food”, “This is a tasty food”, and “This is a healthy food”) into CLIP embedding vectors and calculated the cosine similarity between these query vectors and each food image vector.

From the similarity between linear combination and cosine similarity noted above, we expect that the cosine similarity between the query vectors and the food image vectors can correlate with the ratings from subject experiment. We found that they showed weak correlation with the actual data (correlation coefficients for “like”, “tasty” and “healthy” were r=0.17,0.22,0.33, respectively).

The limited zero-shot performance (r=0.17-0.33) likely reflects the gap between CLIP’s general-purpose training and the specific task of predicting individual food preferences. The higher correlation for “healthy” evaluations (r=0.33) compared to “like” and “tasty” suggests that objective food attributes are more universally captured than subjective preference judgments.

### Prediction/characterization of individual preferences

In this section, we turned to the analysis of individual differences in the rating data. For this purpose, we fitted individual trait vectors for each subject and used these vectors to predict individual preferences.

We applied 5-fold cross-validation and used the averaged value over these folds. The individual preference prediction by CLIP-Emb achieved an average MSE (± standard deviation over subjects) of $1.0\;\pm \;0.3\;\times \;10^{-1}$ (“like”), $1.1\;\pm \;0.4\;\times \;10^{-1}$ (“tasty”), and $9.1\;\pm \;2.8\;\times \;10^{-2}$ (“healthy”) and an average correlation coefficient of 0.674±0.110 (“like”), 0.660±0.108 (“tasty”), and 0.835±0.085 (“healthy”).

We also used different embeddings for the individual preference prediction. From the TEXT-Emb, we obtained average MSE of $1.1\;\pm \;0.4\;\times \;10^{-1}$ (“like”), $1.1\;\pm \;0.4\;\times \;10^{-1}$ (“tasty”), and $9.8\;\pm \;2.9\;\times \;10^{-2}$ (“healthy”) and an average correlation coefficient of 0.642±0.114 (“like”), 0.620±0.116 (“tasty”), and 0.813±0.090 (“healthy”), and from the PIXEL-Emb, we obtained average MSE of $1.4\;\pm \;0.4\;\times \;10^{-1}$ (“like”), $1.4\;\pm \;0.4\;\times \;10^{-1}$ (“tasty”), and $1.7\;\pm \;0.5\;\times \;10^{-1}$ (“healthy”) and an average correlation coefficient of 0.161±0.096 (“like”), 0.190±0.098 (“tasty”), and 0.172±0.057 (“healthy”). From these results, we confirmed that the predictive power of CLIP-Emb yielded better scores for individual rating predictions, compared to TEXT-Emb and PIXEL-Emb.

#### Individual characterization by the preference vector.

**Fig 3:**
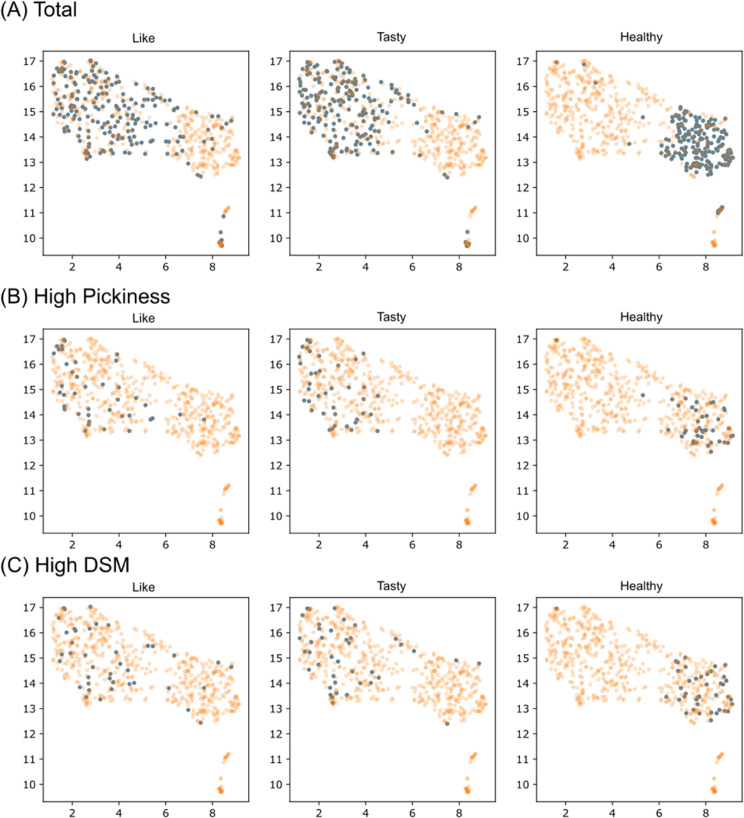
Individual preference vectors visualized in UMAP space. Columns represent preference vectors for ’Like’ (left), ’Tasty’ (middle), and ’Healthy’ (right) attributes. Blue points indicate vectors for the corresponding attribute, while orange points show vectors for other attributes for comparison. Rows display data for different participant groups: (A) all participants, (B) participants with high food pickiness, and (C) participants with high DSM-5 scores.

We analyzed the individual trait vectors $\left(\{x_{i}^{trait}\}\right)$ and the precision ({*r*_*i*_}) obtained through the prediction process to characterize individual differences. First, we visualized all individual trait vectors by UMAP (Fig 3). Each data point corresponds to each subject, and data points from subjects with similar rating patterns appeared closer in the UMAP space.

To compare these individual trait vectors with other individual characterizations, we used the data of questionnaires including the Unbalanced Diet Scale (20 questions) and the DSM-5 Level 1 Cross-Cutting Symptom Measure. We found that people with a high degree of picky eating tend to have “like” and “tasty” vectors that are further away from healthy items. (Fig 3)

**Fig 4:**
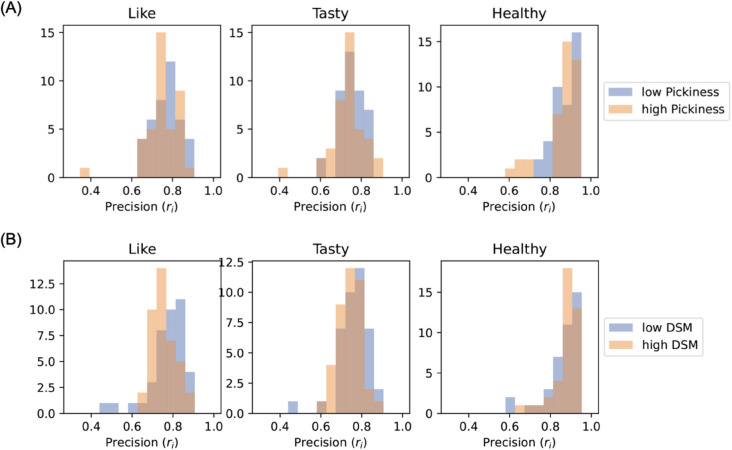
The comparison of distribution of fitting precision ($r_{i}$) for different participant groups. (A) Participants with low / high food pickiness, (B) participants with low / high DSM-5 scores.

On the other hand, while individuals with high DSM scores showed no directional bias in their vectors (see Fig 3), we found that their “like” rating predictions (*r*_*i*_) exhibited significantly lower accuracy (p = 0.024, Mann–Whitney U test; see Fig 4).

## Discussion

In this study, we demonstrated that CLIP embeddings can effectively capture and predict individual food preferences. Our findings provide several important insights into both the methodological aspects of food preference prediction and the underlying mechanisms of preference formation.

### Theoretical implications of CLIP-based food preference prediction

Our results showed that CLIP embeddings (CLIP-Emb) outperformed both pure visual (PIXEL-Emb) and semantic (TEXT-Emb) approaches in predicting food preferences. This superior performance suggests that food preferences emerge from the interplay between visual and semantic features, rather than being determined by either factor alone.

The comparison between CLIP, pixel-based, and text-based embeddings revealed that CLIP and text embeddings showed moderate correlation (r = 0.49), while pixel-based embeddings showed small correlation with both (r = 0.15, 0.10, respectively). These differences in correlation patterns suggest that CLIP embeddings may capture information that is partially shared with text-based representations. While this might suggest a bias towards semantic features, CLIP still maintains sensitivity to visual characteristics. For instance, our analysis suggests (S4 Fig) that CLIP vectors could differentiate between items with identical semantic labels based on their visual attributes, such as different “brownness” of cookies.

### Clinical implications and individual differences

One of our most significant findings was the differential prediction accuracy across different population groups. The decreased prediction accuracy in high-DSM groups, specifically for “like” ratings but not for “tasty” or “healthy” ratings, provides important insights into the nature of food-related decision-making in mental health contexts. Several aspects of this finding warrant detailed discussion.

#### Preference consistency and mental health.

Our analysis revealed an intriguing pattern in the high-DSM group: while their preference ratings showed decreased predictability, their ratings for “tastiness” and “healthiness” maintained consistent predictability. This pattern aligns with previous findings by Strauss et al. [46], who reported that patients with schizophrenia exhibited preference inconsistencies manifested as violations of transitivity in preference ordering (i.e., if A is preferred to B, and B to C, then A should be preferred to C). Since preference ordering inherently involves subjective judgments about personal likes and dislikes, this mechanism would specifically affect subjective preference ratings while leaving objective evaluations intact, which is consistent with our findings.

The absence of directional bias in preference vectors, combined with reduced prediction accuracy, suggests that high-DSM individuals exhibit increased variability in their preference judgments rather than systematic avoidance or approach patterns toward specific food categories. This increased variability may reflect underlying instability in preference formation processes, where individuals struggle to maintain consistent preference criteria across different food items.

From a computational-psychiatry perspective, mood and anxiety disorders have repeatedly been linked to increased choice stochasticity—often formalized as a higher softmax “temperature"—in reinforcement-learning models [47,48]. This account is consistent with our high-DSM result: reduced predictability specifically for “like” ratings indicates lower preference consistency rather than a directional bias.

This selective impairment in preference consistency may be related to aspects of self-concept clarity, as described by Campbell et al. [49]. Personal preferences inherently require integration of sensory evaluation with one’s sense of self and identity, whereas objective judgments about food properties can rely on more universal criteria. The preserved ability to make consistent objective judgments coupled with inconsistent subjective preferences suggests that the challenge lies specifically in the self-referential aspects of preference formation. However, it should be noted that our study was conducted with a non-clinical population, and further research would be needed to establish stronger connections between preference consistency and self-referential processing in clinical contexts.

#### Implications for picky eating behavior.

Our analysis revealed that individuals with high degrees of picky eating tend to have preference vectors that diverge from healthy food items. This systematic bias suggests that picky eating might not just be about food choices but might reflect a more fundamental pattern in how visual and semantic features are integrated into preference formation. This finding has particular relevance for understanding and potentially treating selective eating disorders.

### Methodological considerations and limitations

While our approach shows promise, several methodological considerations and limitations should be addressed.

#### Participant characteristics and generalizability.

Our participant sample, while reasonably balanced in gender distribution (90 males, 109 females), was limited to Japanese adults with a mean age of 39.0 years (SD = 11.4). This demographic composition may limit the generalizability of our findings to other cultural contexts, age groups, and populations with different socioeconomic backgrounds. Food preferences are known to vary significantly across cultures [50,51], and our CLIP-based approach’s effectiveness may differ when applied to participants from different cultural backgrounds or age cohorts. Future studies should investigate the robustness of our methodology across diverse demographic groups, including younger and older adults, and participants from various cultural backgrounds.

#### Dataset constraints.

The Food-Pics_Extended dataset, while extensive, presents several limitations. First, potential cultural biases exist in the image selection, particularly given that our participants were Japanese and some food items might have been less familiar to them. Second, the dataset contained relatively few examples within each food label category, which may have led to an overemphasis on categorical information at the expense of subtle visual variations. Future studies could address this limitation through data augmentation techniques, such as systematic color variations, to better explore the impact of visual features.

Additionally, while our study included measures of picky eating tendencies and mental health indicators, it was limited to a non-clinical population. Future research would benefit from including clinical populations to better understand the full spectrum of food preference patterns.

#### CLIP embedding characteristics.

Our analysis revealed both strengths and limitations of CLIP embeddings in this context. While CLIP successfully captures semantic categories and some visual features like color variations (as demonstrated in S4 Fig with cookie brownness), fine-grained texture information may be underrepresented in the embedding space. This limitation likely stems from CLIP’s training on image-caption pairs where texture descriptions are typically secondary to object identification, leading to embeddings that prioritize semantic categories over subtle visual textures that can influence food preferences.

Several approaches could address these limitations. First, fine-tuning CLIP on food-specific datasets with rich texture annotations could enhance sensitivity to visual properties crucial for preference formation, such as surface texture, freshness indicators, and visual appeal cues. Second, expanding food image datasets to include greater texture and appearance variation within each category would provide richer training signal—for instance, including various preparations of the same food item (crispy vs. soft cookies, rare vs. well-done steak) rather than relying on standard food presentations where texture is largely determined by food category. Third, hybrid approaches combining CLIP’s semantic understanding with specialized texture analysis models could integrate both conceptual and fine-grained visual information. These food-specific adaptations could significantly enhance preference prediction precision while maintaining the multimodal advantages demonstrated in our study.

Furthermore, cultural considerations in CLIP’s training data warrant attention. The model’s training data, predominantly sourced from English-language internet content, likely reflects different cultural backgrounds than our Japanese participant pool. However, the limited zero-shot performance likely reflects multiple factors: the gap between CLIP’s general-purpose training and the specific requirements of preference prediction, as well as cultural differences that may be particularly pronounced for subjective evaluations. This is evidenced by the higher zero-shot correlation for “healthiness” ratings compared to “tasty” and “preference” ratings, suggesting that objective food attributes are more universally represented than culturally-influenced preference judgments.

#### Individual trait vector interpretation.

The individual trait vectors, while effective for prediction, present several conceptual and practical challenges. Beyond the inherent complexity of interpreting high-dimensional vectors, a fundamental limitation lies in our assumption of a shared similarity structure across individuals. In reality, food similarity perceptions likely vary across individuals, suggesting the potential value of personalized embedding spaces - though this would require significantly more data per individual.

Moreover, these preference vectors likely exhibit temporal dynamics, changing with age and experience. To bridge toward real-world clinical applications, longitudinal study designs that track individual trait vectors over time represent a critical next step. Such studies could monitor preference pattern changes during clinical interventions for eating disorders, evaluate the stability of food preference profiles across developmental stages, and assess how preference vectors respond to nutritional counseling or therapeutic interventions.

Specifically, future longitudinal designs should include: (1) repeated measurements of preference vectors at regular intervals (e.g., monthly assessments over 6-12 months) to characterize baseline temporal stability, (2) intervention studies comparing pre- and post-treatment preference patterns in clinical populations, and (3) developmental cohort studies tracking preference vector evolution from adolescence to adulthood. These approaches would establish the clinical utility of our framework for objective monitoring of treatment progress and early detection of preference-related pathology, ultimately enabling personalized dietary interventions based on quantitative preference profiling.

### Further developments

#### Methodological advances.

Future research directions should focus on developing culture-specific embedding models trained on diverse food image datasets, as well as conducting longitudinal studies to track trait vector dynamics across development and intervention periods. Additionally, expanding the analysis framework to incorporate general image preferences beyond food could provide broader insights into preference formation mechanisms. The investigation of visual feature effects through carefully designed experiments would also enhance our understanding of preference formation.

Our preliminary exploration of density-weighted sampling (S1 Text, S7 Fig) suggests potential efficiency gains in data collection. By considering embedding distances during stimulus selection, future studies could optimize their experimental designs, potentially reducing participant burden while maintaining or improving result robustness.

#### Clinical applications.

The differential prediction patterns observed in our study suggest significant potential for clinical applications. The ability to quantify preference patterns through trait vectors could aid in the early detection of eating disorders and provide objective measures for tracking treatment progress. Moreover, understanding individual preference characteristics could guide the development of personalized intervention strategies. The relationship between mental health and food preference formation could be further elucidated through additional research with clinical populations, potentially revealing new approaches to therapeutic intervention.

### Conclusion

This study demonstrates that CLIP embeddings can effectively capture the fundamental structures underlying food preference formation. The observed patterns, particularly in high-DSM groups and picky eaters, suggest that our approach has value not only for practical applications but also for advancing our theoretical understanding of preference formation and decision-making processes.

Beyond food preferences, our framework demonstrates broader applicability rooted in the versatility of vision-language embeddings. Since CLIP can represent diverse object categories beyond food, this approach could extend to other preference domains such as aesthetic preferences for artworks and design elements [52], consumer product evaluations [53], or educational content preferences [54]. More importantly, the shared embedding space enables cross-domain preference comparisons, potentially revealing how individual preference patterns correlate across different categories—for instance, whether someone’s food preferences relate systematically to their aesthetic or product preferences.

The fundamental contribution of our work lies in demonstrating that individual characteristics can be quantified as trait vectors within embedding spaces. When appropriate embeddings capture features relevant to human evaluation, our framework provides a generalizable method for quantifying individual characteristics as trait vectors. This approach enables objective measurement of person-specific patterns across any domain involving human judgment. The trait vector framework has broad implications spanning clinical assessment [55], market research [53], educational personalization [56], environmental policy [57], and beyond. By providing a quantitative foundation for understanding individual differences, this methodology opens new avenues for personalized interventions and evidence-based approaches to human-centered design across diverse domains.

## Supporting information

S1 Text Supplementary Text. Additional technical details supporting the main results, including model comparison analysis, regularization parameter sensitivity analysis, and density-weighted sampling based on embeddings.
(PDF)

S1 Fig Distance matrices from PIXEL-Emb, CLIP-Emb and TEXT-Emb. (TIF)

S2 Fig Classification of points in two dimensional UMAP visualization of CLIP-Emb. (EPS)

S3 Fig Comparison of the UMAP visualizations of three embeddings (PIXEL-Emb, CLIP-Emb and TEXT-Emb). (EPS)

S4 Fig Example of the additional visual features retained in CLIP-Emb, but not in labels. (The labels were “Cookies” for all pictures. The number shown at the top indicates the similarity to the “dark brown” in CLIP-Emb space.) Images are omitted due to licensing; instead, Food_Pics_Extended indices and brief descriptions are shown.
(TIF)

S5 Fig Mean rating visualized on three embeddings (PIXEL-Emb, CLIP-Emb and TEXT-Emb). (EPS)

S6 Fig Comparison between the result of “simulation” in CLIP and actual subjective ratings. (EPS)

S7 Fig Relationship between the number of images used for training and the similarity of the obtained vector to the last vector. (EPS)

S1 Code Analysis Code. (IPYNB)
