# Supplementary material for: Predicting individual food valuation via vision-language embedding model
Source: PLOS Digit Health. 2025 Oct 28;4(10):e0001044. doi: 10.1371/journal.pdig.0001044 (PMC12561901; doi:10.1371/journal.pdig.0001044)
Supplement: S1 Text — Additional technical details supporting the main results, including model comparison analysis, regularization parameter sensitivity analysis, and density-weighted sampling based on embeddings. (PDF) [file pdig.0001044.s001.pdf]

Predicting Individual Food Valuation via Vision-Language Embedding Model: supplemental document

1. MODEL COMPARISON ANALYSIS

To justify our selection of CLIP-ViT for food preference prediction, we conducted a systematic comparison with established vision architectures. The comparison included:

- **CLIP-ViT**: ViT-B/16 backbone; CLIP contrastive pretraining; OpenAI weights [1, 2]
- **CLIP-ResNet**: ResNet-101 backbone; CLIP contrastive pretraining; OpenAI weights [1, 3]
- **ResNet-101**: supervised pretraining on ImageNet-1k [3, 4]
- **ViT-B/16**: supervised pretraining on ImageNet-21k [2, 4]
- **EfficientNet-B0**: supervised pretraining on ImageNet-1k [4, 5]

All models were evaluated under two conditions: with intercept terms (intercept=True) to optimize each method's performance, and without intercept terms (intercept=False) to maintain consistency with cosine similarity interpretation. For subsequent analyses, we adopted the intercept=False configuration to enable direct geometric interpretation of preference patterns in embedding space, where the linear model $\mathbf{y} = \mathbf{X}\boldsymbol{\beta}$ corresponds to cosine similarity between preference vectors $\boldsymbol{\beta}$ and image embeddings $\mathbf{X}$.

Table S1. Performance comparison across different vision architectures for average rating prediction using 5-fold cross-validation.

Method	Rating	Intercept=False		Intercept=True	
		Test Corr	Test MSE	Test Corr	Test MSE
CLIP-ViT	Like	0.771 ± 0.019	0.039 ± 0.002	0.775 ± 0.021	0.039 ± 0.003
	Tasty	0.792 ± 0.014	0.041 ± 0.002	0.794 ± 0.016	0.041 ± 0.002
	Healthy	0.944 ± 0.006	0.048 ± 0.002	0.945 ± 0.006	0.047 ± 0.003
CLIP-ResNet	Like	0.748 ± 0.024	0.040 ± 0.002	0.748 ± 0.024	0.041 ± 0.002
	Tasty	0.770 ± 0.024	0.043 ± 0.001	0.770 ± 0.026	0.043 ± 0.001
	Healthy	0.928 ± 0.004	0.054 ± 0.003	0.928 ± 0.004	0.054 ± 0.003
ResNet-101	Like	0.105 ± 0.051	2.241 ± 0.049	0.520 ± 0.035	0.059 ± 0.002
	Tasty	0.073 ± 0.071	2.260 ± 0.050	0.558 ± 0.037	0.062 ± 0.001
	Healthy	0.218 ± 0.077	2.018 ± 0.039	0.780 ± 0.029	0.094 ± 0.004
ViT-B/16	Like	0.153 ± 0.113	2.393 ± 0.175	0.482 ± 0.045	0.079 ± 0.002
	Tasty	0.123 ± 0.110	2.415 ± 0.179	0.524 ± 0.038	0.084 ± 0.003
	Healthy	0.190 ± 0.083	2.153 ± 0.156	0.768 ± 0.014	0.108 ± 0.004
EfficientNet-B0	Like	0.097 ± 0.042	2.312 ± 0.052	0.472 ± 0.031	0.066 ± 0.002
	Tasty	0.056 ± 0.047	2.331 ± 0.052	0.530 ± 0.036	0.069 ± 0.003
	Healthy	0.211 ± 0.069	2.085 ± 0.047	0.708 ± 0.020	0.111 ± 0.004

The results demonstrate the clear superiority of CLIP-based methods, particularly CLIP-ViT, which achieved the highest correlation coefficients across all rating dimensions. The superior performance of multimodal CLIP architectures compared to unimodal vision models highlights the importance of integrating both visual and semantic information for food preference prediction.

2. REGULARIZATION PARAMETER SENSITIVITY ANALYSIS

To select an appropriate regularization parameter, we evaluated ridge regression performance across $\lambda \in [0, 0.1, 0.2, 0.3, 0.4, 0.5, 0.6, 0.7, 0.8, 0.9, 1.0, 1.2, 1.4, 1.6]$ using the same 5-fold cross-validation framework employed for final evaluation. Table S2 shows the train and test correlations for average rating prediction (like dimension) across different λ values.

Table S2. Ridge regression performance across regularization parameters

λ	Train r ($\pm$SD)	Test r ($\pm$SD)	Train-Test Gap
0.0	0.970 $\pm$ 0.001	0.608 $\pm$ 0.039	0.362
0.1	0.906 $\pm$ 0.002	0.798 $\pm$ 0.017	0.108
0.2	0.887 $\pm$ 0.002	0.796 $\pm$ 0.017	0.091
0.3	0.874 $\pm$ 0.003	0.793 $\pm$ 0.017	0.081
0.4	0.864 $\pm$ 0.003	0.789 $\pm$ 0.018	0.075
0.5	0.856 $\pm$ 0.003	0.785 $\pm$ 0.018	0.071
0.6	0.849 $\pm$ 0.003	0.782 $\pm$ 0.018	0.067
0.7	0.844 $\pm$ 0.003	0.779 $\pm$ 0.019	0.065
0.8	0.838 $\pm$ 0.003	0.776 $\pm$ 0.019	0.062
0.9	0.834 $\pm$ 0.003	0.774 $\pm$ 0.019	0.060
1.0	0.829 $\pm$ 0.004	0.771 $\pm$ 0.019	0.058
1.2	0.822 $\pm$ 0.004	0.767 $\pm$ 0.019	0.055
1.4	0.815 $\pm$ 0.004	0.763 $\pm$ 0.020	0.052
1.6	0.809 $\pm$ 0.004	0.759 $\pm$ 0.020	0.050

The analysis revealed severe overfitting at $\lambda = 0$ (train-test gap = 0.362) and peak test performance at $\lambda = 0.1$–0.2 ($r \approx 0.798$). Applying the one-standard-error rule to the peak performance at $\lambda = 0.1$ (0.798 $\pm$ 0.017) yields a threshold of 0.781, suggesting $\lambda \approx 0.7$ as the most regularized parameter within one standard error of optimal performance.

However, given our focus on individual trait vector characterization and the moderate sample size ($n = 199$), we selected $\lambda = 1.0$ as a conservative choice that ensures stable coefficient estimation for subsequent psychological analysis. While this results in a modest reduction in test performance ($\Delta r = 0.027$ compared to peak), the improved train-test balance (gap = 0.058) and enhanced coefficient stability justify this conservative approach for our analytical framework.

3. DENSITY-WEIGHTED SAMPLING BASED ON THE EMBEDDING

We applied the density-weighted sampling method to the training process. We calculated kNN for each food image and defined the density of each point as the distance to this k-th nearest neighbor. In the density-weighted sampling process, we sampled data points with probability proportional to the inverse density. We compared the training results with and without density-weighted sampling. We found that this process slightly enhanced the convergence to a particular vector (Fig S7).

In the main manuscript, we used the vector obtained from fitting the full dataset for the characterization. The similarity exceeds 0.9 when the train ratio was around 0.4, so the characterization described below was not sensitive to the choice of the data used for fitting.

REFERENCES

1. A. Radford, J. W. Kim, C. Hallacy, *et al.*, “Learning transferable visual models from natural language supervision,” in *International conference on machine learning,* (PMLR, 2021), pp. 8748–8763.
2. A. Dosovitskiy, L. Beyer, A. Kolesnikov, *et al.*, “An image is worth 16x16 words: Transformers for image recognition at scale,” arXiv preprint arXiv:2010.11929 (2020).
3. K. He, X. Zhang, S. Ren, and J. Sun, “Deep residual learning for image recognition,” in *Proceedings of the IEEE conference on computer vision and pattern recognition,* (2016), pp. 770–778.
4. J. Deng, W. Dong, R. Socher, *et al.*, “Imagenet: A large-scale hierarchical image database,” in *2009 IEEE conference on computer vision and pattern recognition,* (Ieee, 2009), pp. 248–255.
5. M. Tan and Q. Le, “Efficientnet: Rethinking model scaling for convolutional neural networks,” in *International conference on machine learning,* (PMLR, 2019), pp. 6105–6114.
