# Supplementary figures and images for: Predicting individual food valuation via vision-language embedding model
Source: PLOS Digit Health. 2025 Oct 28;4(10):e0001044. doi: 10.1371/journal.pdig.0001044 (PMC12561901; doi:10.1371/journal.pdig.0001044)

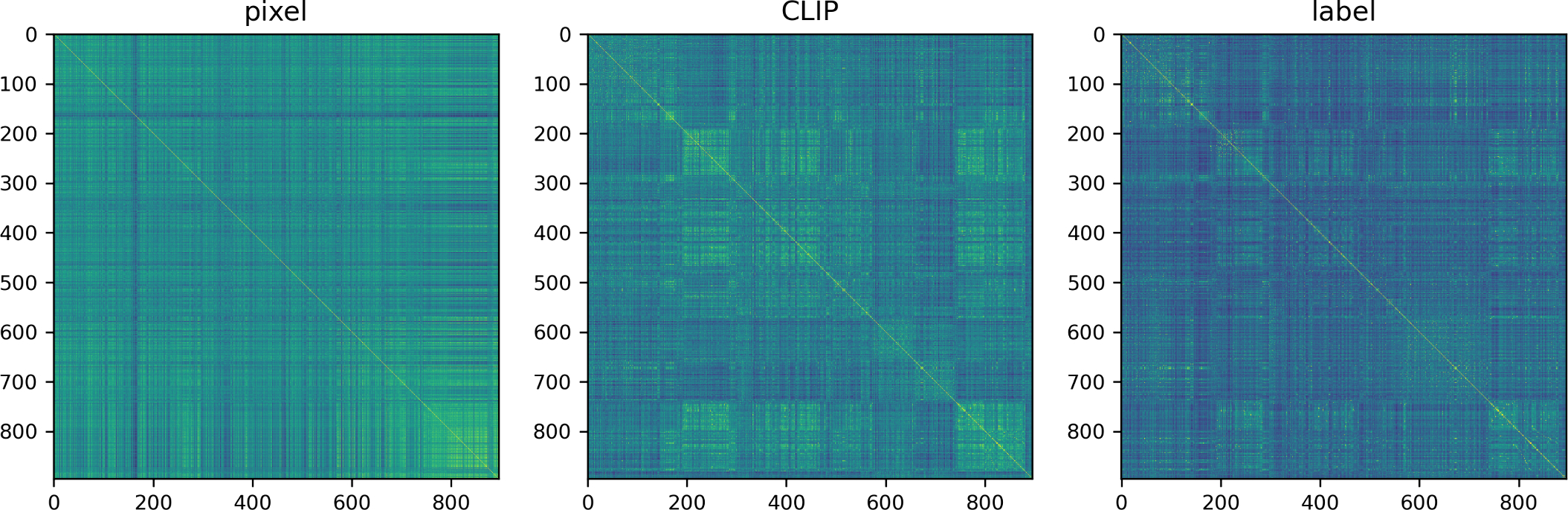

Supplement: S1 Fig — (TIF) [file pdig.0001044.s002.tif]

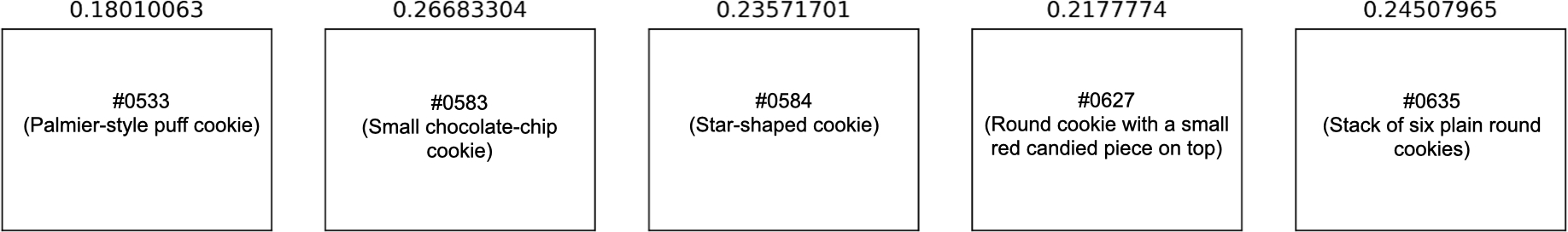

Supplement: S4 Fig — (The labels were “Cookies” for all pictures. The number shown at the top indicates the similarity to the “dark brown” in CLIP-Emb space.) Images are omitted due to licensing; instead, Food_Pics_Extended indices and brief descriptions are shown. (TIF) [file pdig.0001044.s005.tif]
